# Complete Genome Sequence of a Phage Infecting *Sphingomonadaceae*

**DOI:** 10.1128/mra.00366-22

**Published:** 2022-06-02

**Authors:** Shang Shen, Tetsunobu Anazawa, Tomonari Matsuda, Yoshihisa Shimizu

**Affiliations:** a Department of Engineering, Kyoto University, Otsu, Shiga, Japan; b Lake Biwa Branch Office, National Institute for Environmental Studies, Otsu, Shiga, Japan; Portland State University

## Abstract

We isolated a phage infecting a member of the *Sphingomonadaceae* family from a freshwater lake. The phage has a DNA genome of 41,771 bp, with a GC content of 61.7%. The genome harbors 50 predicted protein-coding genes and an auxiliary metabolic gene, which encodes a protein belonging to the radical *S*-adenosylmethionine superfamily.

## ANNOUNCEMENT

Members of the family *Sphingomonadaceae* play important roles in aquatic environments (1–4). However, little is known about the phages infecting *Sphingomonadaceae* and how they control the abundance of this family.

The phage, infecting a member of the *Sphingomonadaceae* family, was isolated from a water sample collected from the surface of a freshwater lake (Lake Biwa, Japan; 35°23′21.0″N, 136°07′51.0″E) and from a strain of *Sphingomonadaceae* family (BSN-002 [deposited in GenBank under accession number CP091804]) (5). The collected lake water was filtered through 0.2-μm-pore-size polycarbonate filters. The filtrates and the preincubated host strain were incubated using the double-agar method at 25°C for 2 weeks. Virions contained in the plaques were purified twice using the same method. Phage DNA was extracted using the DNeasy blood and tissue kit (Qiagen, Hilden, Germany). The library was prepared using a Nextera XT DNA sample preparation kit (Illumina, San Diego, CA) according to the manufacturer’s protocol. The sample was then sequenced using a MiSeq sequencing system with a V3 (2× 300-bp reads) reagent kit (Illumina). Bioinformatic analysis was performed as described below, and default parameters were used for all software unless otherwise specified. Raw reads (524,664 paired-end reads) with low-quality regions were removed using Trimmomatic with default settings (v.0.39) (6), and finally, 515,345 paired-end reads were obtained. The phage genome was assembled using SPAdes with the –careful option (v.3.13.1) (7) and identified as a complete (i.e., circular) genome using ccfind (v.1.1) (8). Open reading frames (ORFs) were predicted using Prodigal with the -p meta option (v.2.6.3) (9) and annotated by eggNOG-Mapper v.1.0.3 (10) using the -m diamond option. ORFs were also annotated using HMMER v.3.1b2 software (11) against the Prokaryotic Virus Orthologous Groups databases (12) and using the highly sensitive HMM-HMM search (8) against Pfam v.31.0 databases using HHsearch (13) and JackHMMER (14).

The genome of the phage (VSN-002) infecting a member of the *Sphingomonadaceae* family was found to comprise 41,771 bp, with a GC content of 61.7%. Of the 75 predicted ORFs, 41 were leftward oriented, 65 started with ATG, 8 started with GTG, and 2 started with TTG (Table 1). A total of 50 proteins were encoded in the phage genome, varying in length from 138 to 3,186 bp. Of the 50 proteins, 19 were classified as hypothetical or uncharacterized proteins, and 31 were assigned putative functions. The phage genome was found to encode proteins related to phage replication (e.g., terminase, DNA helicase, capsid, and tail). An auxiliary metabolic gene (AMG), which may be expressed during infection to increase viral production by promoting host metabolism, was also found in the phage genome. The phage genome was found to have a radical *S*-adenosylmethionine (SAM) superfamily that may be involved in critical roles in numerous biosynthetic pathways in bacteria (e.g., synthesis of protein cofactors) (15). BLASTn analysis showed that our *Sphingomonadaceae* phage was not homologous (more than 95% similarity) to previously reported bacteriophages, and the host of this phage was also not assigned to any existing species in the Genome Taxonomy Databases (5).

**TABLE 1:** Summary of predicted open reading frames and their annotation

Parameter	Value
Length of genome (bp)	41,771
GC content (%)	61.7
No. of predicted ORFs	
Total	75
Leftward	41
Rightward	34
Start codon with ATG	65
Start codon with GTG	8
Start codon with TTG	2
No. of ORFs annotated	
Total	50
Hypothetical or uncharacterized proteins	19
Putative functions	31

### Data availability.

The complete bacteriophage genome has been deposited in GenBank (accession number MZ127829). Raw reads from whole-genome sequencing data were deposited in the DDBJ (accession number DRR361504).
